# Modification and optimization of an established prognostic score after re-irradiation of recurrent glioma

**DOI:** 10.1371/journal.pone.0180457

**Published:** 2017-07-05

**Authors:** Kerstin A. Kessel, Josefine Hesse, Christoph Straube, Claus Zimmer, Friederike Schmidt-Graf, Jürgen Schlegel, Bernhard Meyer, Stephanie E. Combs

**Affiliations:** 1 Department of Radiation Oncology, Technische Universität München (TUM), Munich, Germany; 2 Institute of Innovative Radiotherapy (*i*RT), Helmholtz Zentrum München, Neuherberg, Germany; 3 Department of Neuroradiology, Technical University of Munich (TUM), Munich, Germany; 4 Department of Neurology, Technical University of Munich (TUM), Munich, Germany; 5 Department of Neuropathology, Technical University of Munich (TUM), Munich, Germany; 6 Department of Neurosurgery, Technical University of Munich (TUM), Munich, Germany; National Institutes of Health, UNITED STATES

## Abstract

**Introduction:**

For about 30 years, researchers developed prognostic scores and searched for prognostic factors to predict outcomes for cancer patients. The “Combs Prognostic Score” for re-irradiation in recurrent glioma was recently validated and results showed that the score is a significant (p < .001) and reliable predictor for patients undergoing re-irradiation (re-RT). We sought to enhance the score and generated a novel scoring approach, taking into account the information on resection of recurrent tumors, KPS, and tumor volume.

**Patients and methods:**

The prognostic score was generated based on 209 patients treated between 2002 and 2016 for recurrent glioma at the department of radiation oncology at the Klinikum rechts der Isar, Munich. To further enhance the previously validated Combs Prognostic Score, which uses the prognostic factors primary histology, time between primary RT and re-RT, and age, we added KPS, tumor volume (PTV) and re-resection into the scoring scheme.

**Results:**

The median follow-up time was 3.5 months. 67.5% were WHO IV gliomas with a median OS after re-RT of 7.9 months, 17.7% were WHO III gliomas with an OS of 11.3 months and 14.8% were WHO I/II gliomas with an OS of 14.7 months. Multivariate analyses confirmed the prognostic factors KPS (p < .001) and showed a tendency to significance for tumor volume (p = .067) and re-resection (p = .064). The new prognostic score demonstrated a high significance (p < .001).

**Conclusion:**

The “New Combs Prognostics Score” is a significant and useful tool to predict the overall effect of re-RT in patients with recurrence gliomas. This modified score offers an even better way to classify patients in clinical routine and prospective clinical trials investigating re-irradiation.

## Introduction

For about 30 years, researchers developed prognostic scores and searched for prognostic factors to predict outcomes for cancer patients. Each scoring system may help with treatment decision making and includes different tumor and patient parameters. Combs et al. [1] published the first prognostic score for re-irradiation in recurrent glioma including patient age, histology and the time between first and second radiotherapy (RT) as key factors. One of the main difficulties in establishing and validating a score is the fact of patient heterogeneity, such as various histologies, time intervals between primary and secondary RT, different target volume concepts, and different time points during the course of the disease. To validate the “Combs Prognostic Score” efficacy we recently analyzed the significance using a different, independent and relatively homogeneous patient cohort [2]. Results showed that the “Combs Prognostic Score” is a significant and reliable predictor (p < .001) for patients with recurrent glioma undergoing re-irradiation (re-RT).

While the real value of re-RT is discussed controversially in recurrent gliomas [3,4], the identification of prognostic and predictive factors becomes increasingly important [5]. Until today, no standard treatment has been defined in the case of high-grade glioma recurrence. Age at treatment, primary histology, tumor volume, Karnofsky Performance Score (KPS), re-resection, and the time between primary and re-RT are known to have an influence on outcome, however, different research groups generated varying results in terms of their significance [6–10]. Recent evaluations analyzed the prognostic impact of the O6-Methylguanin-DNA-Methyltransferase (MGMT)-promotor status [11], resection [12,13], and radiation dose [2]. MGMT-promotor methylation is one of the strongest predictors of outcome, however, methylation status might change between initial diagnosis and recurrence. Since tumor tissue is often not available at recurrence, the integration of molecular markers in such as prognostic score might limit the wide use in clinical routine [14].

Based on our previously established score, we learned that there is an increasing role of surgery in the treatment of recurrent glioma [15]. Ringel et al. [12] could show in a multicenter study that patients benefit from re-resection in the recurrent setting. Many patients, however, are not fit for re-resection, or the tumor complexity does not allow for a total resection.

To enhance the “Combs Prognostic Score” we extended the scoring approach, taking into account the information on previous surgical procedures, KPS and tumor volume. In the present manuscript, we optimized and modified the score to define patients more precisely which benefit from a second course of RT.

## Materials and methods

### Patients

From 2002 to 2016 a total of 209 patients with recurrent glioma were consecutively treated at the department of radiation oncology at the Klinikum rechts der Isar, Munich. Table 1 describes the detailed patient characteristics. Adjuvant chemotherapy temozolomide (TMZ) was prescribed when indicated.

**Table 1:** Patients characteristic.

	Patients, n (%)
Gender	
Female	90 (43.0)
Male	119 (57.0)
Age at re-RT (median, range) [years]	55 (21–79)
Glioma histology at diagnosis	
WHO I/II	31 (14.8)
WHO III	37 (17.7)
WHO IV	141 (67.5)
Glioma histology at recurrence	
WHO II	4 (1.9)
WHO III	46 (22.0)
WHO IV	159 (76.0)
PTV (median, range) [ml]	49.3 (0.4–480.6)
>47 ml	107 (51.2)
<= 47 ml	102 (48.8)
KPS	
>= 80%	113 (54.1)
< 80%	96 (45.9)
MGMT-promotor * status	
Methylated	37 (17.7)
Not methylated	50 (23.9)
Unknown	122 (58.4)
Number of surgeries between primary RT and re-RT	
0	99 (47.4)
1	82 (39.2)
≥2	28 (13.4)
Resection status	
Complete	32 (15.3)
Incomplete/unknown	78 (37.3)
Time from re-resection to re-RT, (median, range) [months]	
WHO III	1.66 (0.62–20.89)
WHO IV	1.61 (0.39–33.48)
Time between primary RT and re-RT, (median, range) [months]	
WHO III	32.85 (6.70–228.10)
WHO IV	14.70 (1.00–222.90)

* O6-Methylguanin-DNA-Methyltransferase

Data were retrospectively obtained from the clinical information systems and analyzed anonymously, therefore, written informed consent was not necessary. The nature and content of the study were approved by the Ethics Committee of the Technical University of Munich (TUM) (project number 408/14).

### Treatment and follow-up

Patients received a primary RT of median 60 Gy (range 40–66 Gy, single dose 1.8/2 Gy) and within a median time of 18 months a secondary RT of median 30 Gy (range 14–60 Gy, single dose 1.8/2 Gy) using either radiosurgery, 3D, intensity modulated radiotherapy (IMRT) or stereotactic fractionated radiotherapy (FSRT) technique. Treatment decision for re-RT was made in interdisciplinary tumor boards. Treatment planning was based on CT, MRI (T1) and aminoacid PET when available. The median planning target volume (PTV) was 49.3 ml (range 0.4–480.6 ml).

Of all, 110 (52.6%) patients received re-resection due to tumor progression, 32 (15.3%) as complete resection.

Follow-up included contrast-enhanced MR-imaging and clinical assessment and was performed every 2–3 months. Additional examinations were scheduled as clinically needed.

### Score calculation

We enhanced the previously validated Combs Prognostic Score [2], which uses the prognostic factors: primary histology, time between primary RT and re-RT, and age, and added KPS, tumor volume (PTV) and re-resection into the scoring scheme (Table 2). MGMT-promotor methylation was not included as it was only available for 87 (41.6%) patients and we are aware that it is not always available in routine treatment and could even change during the course of the disease.

**Table 2:** Scoring scheme of the new prognostic score (modification of the original scoring scheme by Combs et al. [1]).

Prognostic factor	Prognostic value
Primary histology	
Glioblastoma, WHO IV	2
Anaplastic glioma, WHO III	1
Low-grade glioma, WHO I/II	0
Age	
>= 50 years	1
< 50 years	0
Time between primary radiotherapy and Re-irradiation	
<= 12 months	1
> 12 months	0
Re-resection performed	
no	1
yes	0
KPS	
< 80%	1
>= 80%	0
Tumor volume (PTV)	
> 47 ml	1
<= 47 ml	0

### Statistics

Statistical calculations were performed using SPSS Statistics v23 (IBM, USA). Overall survival (OS) was calculated from the first day of re-irradiation until death or last follow-up. Survival analyses were based on the Cox regression method. A p-value ≤ 0.05 was considered as statistically significant.

## Results

### Outcome and univariate analyses

The median follow-up time was 3.5 (95% CI: 4.7–7.4) months. 67.5% were WHO IV gliomas with a median OS after re-RT of 7.9 (95% CI: 6.8–8.8) months, 17.7% were WHO III gliomas with an OS of 11.3 (95% CI: 7.8–14.9) months and 14.8% were WHO I/II gliomas with an OS of 14.7 (95% CI: 10.6–18.8) months.

Table 3 shows the results of both the univariate and multivariate analyses including the prognostic factors: Primary histology, age, time between primary RT and re-RT, KPS, re-resection, neurological symptoms, PTV volume and dose group.

**Table 3:** New prognostic scoring groups.

Scoring group	Scoring value
a	0–1
b	2–3
c	4–5
d	6–7

To account for the generally offered re-resection before re-RT, we compared OS in both groups (re-resection performed yes vs. no). The difference was significant at p = .013 (HR: 0.69; 95% CI: 0.51–0.93) and with p = .043 if we analyze only patients with re-resections (n = 110, 52.6%) separated by extent of resection (EOR) (complete vs. non-complete resection). EOR was defined by the surgeons based on early post-surgery imaging.

### Modification of the Combs prognostic score

**Fig 1:**
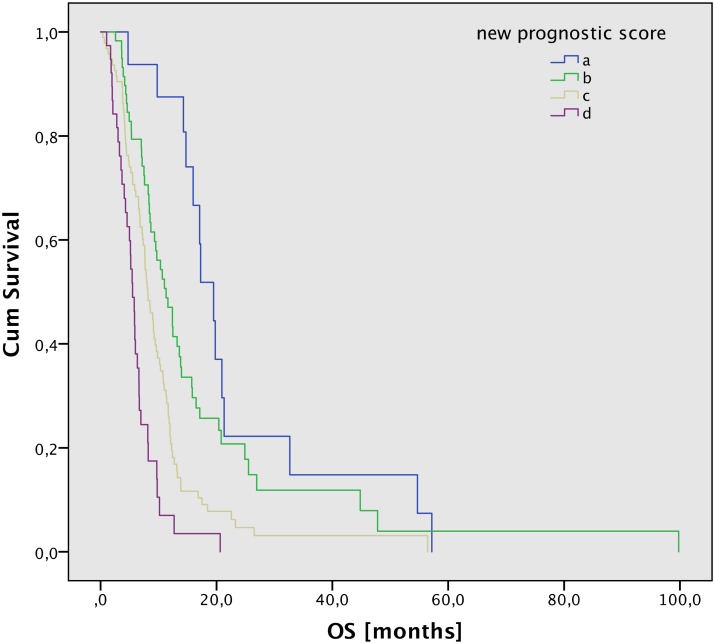
OS after according to the new prognostic score (p < .001).

In the multivariate analyses KPS, dose group, and MGMT were significant; PTV and re-resection were borderline significant (Table 4). MGMT and dose group were not included in the score modification, as the MGMT information was only available for a subgroup of patients and the dose group distribution was very inhomogeneous. Accordingly, we developed an enhanced scoring scheme by adding KPS, re-resection, and PTV as new prognostic factors (Table 2). With the new scoring scheme, a new scoring value from 0–8 would be possible. We grouped them and built four new scoring groups, see Table 3. The new score showed a high significance on OS with p < .001 (Fig 1). Median OS and life tables are listed in Table 5.

**Table 4:** Results of the univariate and multivariate analyses of the prognostic factors.

		Univariate analyses			Multivariate analyses		
		HR	95% CI	p-value	HR	95% CI	p-value
Primary histology	WHO I/II **			.001 *			.1
	WHO III	0.45	0.29–0.70		0.31	0.05–1.95	
	WHO IV	0.65	0.43–0.96		0.36	0.10–1.27	
Age (≥50y vs. <50y)		1.64	1.19–2.25	.002 *	1.32	0.48–3.68	.2
Time between primary RT and re-RT (>12m vs ≤12m)		2.08	1.51–2.86	< .001 *	1.01	0.37–2.74	.3
KPS (≥80% vs. <80%)		1.94	1.44–2.62	< .001 *	3.27	1.35–7.95	< .001 *
Neurological symptoms (yes vs. no)		1.21	0.90–1.64	.2	0.70	0.34–1.42	.1
Gender (male vs. female)		0.98	0.73–1.32	.9	0.94	0.65–1.36	.5
PTV volume (≥47ml vs. <47ml)		1.18	0.88–1.59	.3	1.89	1.25–2.85	.067
Dose group ***	A **			.1			.002 *
	B	1.58	0.87–2.88		1.84	0.90–3.75	
	C	1.40	0.87–2.25		0.42	0.21–0.81	
	D	1.99	1.16–3.41		1.83	1.04–3.22	
MGMT-promotor (methylated vs. not methylated)		3.56	2.0–6.32	.001 *	1.91	1.16–3.17	.007 *
Re-resection (yes vs. no)		0.69	0.51–0.93	.013 *	2.34	1.45–3.79	.064

HR = Hazard Ratio; 95% CI = 95% Confidence Interval;

* = Significant p-value;

** = Reference group of categorical variable;

*** dose in EQD2 (a/ß = 10Gy) [Gy] A: < = 36 Gy (mainly 36 à 2 Gy), B: >36 Gy < = 38 Gy (only 30 à 5 Gy), C: >38 Gy < = 40 Gy (only 36 à 3 Gy), D: >40 Gy (mainly 46 à 2 Gy).

**Table 5:** New prognostic score: Median OS and life table.

New score	Number of patients	Median OS	Proportion surviving after			
			6 months	12 months	24 months	36 months
a	16	19.5	94%	88%	22%	15%
b	60	11.3	79%	47%	21%	12%
c	95	8.1	70%	22%	5%	3%
d	38	5.5	41%	7%	0%	0%

## Discussion

In our analysis, we modified the “Combs Prognostic Score” [1] by means of data from 209 glioma patients. All patients were treated consecutively with re-irradiation in a single center. Previously, the results of the former score have been confirmed and validated [2]. Since neurosurgical resection can be of value in the recurrent setting, we modified the scoring system including not only primary histology, age and time between primary RT and re-RT but also the performance of re-resection between radiotherapies as well as KPS and tumor volume (PTV). The new prognostic score demonstrated a high significance (p < .001).

Until today, no standard treatment for glioma patients with recurrent disease exists. Possible treatment options depend on patient characterizing factors, such as age, KPS, tumor size, and time of recurrence. If safely possible, re-resection should be offered to patients, and several groups have shown that especially the extent of resection (EOR) has a significant impact on survival [12]. Moreover, the combination of recurrence resection and any other additional treatment are more effective than resection followed by a wait-and-see strategy; generally, treatment decisions are made based on the tumor burden after surgery. In cases of alleged complete resection, re-RT is commonly withheld, and chemotherapy or other systemic agents, i.e. bevacizumab or other molecular targeted treatments, are offered [16]. In cases of macroscopic tumor residuals, re-RT is offered in many centers.

While early trials of re-RT offered only modest doses due to the fear of treatment-related side effects, modern techniques offer the possibility to deliver higher doses [17–20]. A dose-response relationship has been shown by several groups; also, data from the present manuscript demonstrate that higher doses lead to increase survival times [21]. However, dose application is dependent on many factors, i.e. time between first and second radiotherapy, volume, as well as other pretreatment and patient-individual factors. Thus, a set dose recommendation may be difficult. Moreover, an associated with higher single doses and the rate of symptomatic necrosis has been shown; therefore, strongly hypofractionated regimens or radiosurgery in larger volumes should be applied with caution [18,20,22,23].

The search for the optimal treatment strategy in recurrent gliomas in ongoing: After the development of a first prognostic score to predict outcome [1], many groups have tried to develop such tools, taking into account several factors, some already known as prognostic factors, some recently discovered ([6,8,12]). Molecular determinant, such as MGMT or 1p19q deletions have been shown to be predictive for outcome, thus further insight into molecular architectures is currently sought for. The integration of molecular tumor markers and microRNA profiles into the interdisciplinary treatment decision is a relatively new field [14,24]. They are currently evaluated if they can change the diagnosis and therapy concepts of glioma patients. Two works from Hayes et al. [25,26] showed promising results. They analyzed the microRNA profile of 51 patients treated with bevacizumab and found a correlation between OS. Sana et al. [27] developed a risk score based on miRNA expression and showed significant influence on survival as well (p < .001).

However, it must be kept in mind that recent tumor histology must be available if molecular determinants are taken into account. This means tumor tissue must be available from the recurrent situation [14] and in most cases—about 60% of our cohort—it is not available. Acquiring additional tumor specimens must be justified by a valid and evident treatment benefit for the patient since every intervention can be associated with additional side effects. Molecular analyses are cost and time intensive and it is not yet clear which of the molecular analyses are ideal. The integration of molecular markers in a prognostic score might limit the wide use in clinical routine and needs further evaluations.

Only recently arguments are emerging arguing for re-resection as it offers the best possible outcome followed by adjuvant re-irradiation and/or chemotherapy if indicated [5]. Since the EOR is significantly associated with survival, this factor should be taken into account, when safely possible. Our current evaluations showed that EOR has a significant impact on survival. Perhaps, in the future, re-operation and early re-RT, independently of macroscopic tumor, can increase the effect of re-RT and prolong survival times. This concept is currently being evaluated in a randomized prospective clinical trial (GlioCAVE/NOA 17).

## Conclusion

The “New Combs Prognostics Score” is a significant and useful tool to predict the overall effect of re-RT in patients with recurrent gliomas. Compared to the previous score, the role of KPS, tumor volume and re-resection are taken into account. Molecular markers were not included since uncertainties in their role in recurrence still exist, and often tumor tissue of recurrent tumors is not available for fast integration into decision making. In conclusion, on the example of re-RT in recurrent glioma, we generated a prognostic score, which takes into account patients’ characteristics as well as neurosurgical interventions: This modified score offers an even better way to classify patients in clinical routine and prospective clinical trials investigating re-irradiation.
